# Accreditation of pharmacy programs and its impact on SPLE success and pharmacist readiness in Saudi Arabia

**DOI:** 10.3389/fmed.2024.1490555

**Published:** 2024-11-07

**Authors:** Dalia Almaghaslah

**Affiliations:** Clinical Pharmacy Department, College of Pharmacy, King Khalid University, Abha, Saudi Arabia

**Keywords:** SPLE, pharmacy, Saudi Arabia, accreditation, NAPLEX, licensure

## Abstract

**Aim:**

The impact of pharmacy program accreditation on the Saudi Pharmacists Licensure Examination (SPLE) pass rates and overall pharmacist readiness was investigated.

**Methods:**

A cross-sectional retrospective study was conducted. Data on SPLE pass rates were obtained from the Saudi Commission for Health Specialties (SCFHS) 2024 report. Pharmacy colleges were categorized into five groups based on their students' average SPLE scores. Information on the national i.e., the Evaluation and Training Evaluation Center (ETEC) and international i.e., the American Council for Pharmacy Education (ACPE) and the Canadian Council for Accreditation of Pharmacy Programs (CCAPP) accreditation status of these colleges was also collected.

**Results:**

Higher average SPLE scores (mean = 563, SE = 43.4) were observed in accredited colleges (either national or international) compared to non-accredited colleges (mean = 533, SE = 33.6), with a significant difference noted [*t*_(22)_ = −2.149, *p* = 0.042]. Higher average SPLE scores (mean = 581.8, SE = 18.9) were also found in colleges with multiple accreditations compared to those with fewer or no accreditations (mean = 548.02, SE = 18.9), though this difference was not statistically significant [*t*_(25)_ = −1.8, *p* = 0.086].

**Discussion and conclusion:**

It was demonstrated that accreditation, whether national or international, is associated with higher SPLE pass rates, indicating a positive impact on exam performance. National accreditation by ETEC alone was found to be sufficient for improving SPLE scores and ensuring pharmacist readiness, whereas dual or international accreditations did not provide additional benefits in this context.

## Introduction

Delivery of high-quality health services is heavily reliant on the competencies of the health workforce who deliver these services (16). Factors affecting the quality of health workforce performance vary. Some factors are linked to the scope and length of pre-professional education and training. Education programs are evaluated to meet educational standards and qualifications through national and international accreditation authorities, whereas individuals are assessed through licensure (1). A competent health workforce attains the identified and agreed-upon knowledge, skills, behaviors, and values by regulatory bodies. Competency in the pharmacy context refers to the knowledge, skills, attitudes, and behaviors that an individual develops through education, training, and experience (17). Governance of the pharmacy profession is different in countries around the world; however, there is consensus on the registration (licensing) of pharmacists before they seek employment within their profession. Other regulatory mechanisms are engaged in the accreditation of pharmacy professional programs and continuing professional development (2, 15). There are no universal standards for pharmacist licensure (15). Some credentialing authorities mandate that qualified pharmacists pass a written examination, oral assessment, and/or Objective Structured Clinical Examination (OSCE) to obtain licensing (15). Other nations oblige pharmacists to complete practical placement or clerkship in addition to a professional licensing examination as a requirement for registration. In contrast, other countries limit registration criteria to earning an educational degree and payment of registration charges.

Regulating the pharmacy profession in Saudi Arabia is relatively similar to those implemented in developed countries. It begins with mandating accreditation or certification of pharmacy education programs as well as licensing of eligible pharmacists seeking patient-facing roles in pharmacy sectors (3). National accreditation was first implemented in 2018 by the Education and Training Evaluation Commission (ETEC) through setting academic standards for professional degrees in pharmacy (4). However, not all colleges have achieved or maintained accreditation. These standards play an important role in establishing the minimum curriculum requirements for first degrees in pharmacy, PharmD/BS in pharmacy. They also guarantee the academic quality of the programs and ensure their capability to graduate competent pharmacy professionals. A need-based approach is another crucial criterion for designing a curriculum. A minimum of 160 credits is required and is distributed as follows: 11% Biomedical Sciences, 10% Pharmaceutics, 7% Pharmacology, 6% Medicinal Chemistry, 15% Clinical Pharmacy Sciences, 16% Pharmacotherapy, 4% Pharmaceutical Research, and 33% Experiential Training (5).

The Saudi Pharmacists Licensure Examination (SPLE) was made a requirement for pharmacist licensure by the Saudi Commission for Healthcare Specialties in 2019 for pharmacists graduating from local institutions (18). The examination covers four areas: 10% Basic Biomedical Sciences, 35% Pharmaceutical Sciences, 20% Social/Behavioral/Administrative, and 20% Clinical Sciences (13). The exam consists of two parts, each containing 100 multiple-choice questions (MCQs) and allowing 120 min for completion (14). A passing score on the exam is achieved with a score > 536 on a scale of 200–800. Pharmacy colleges' pass rates have been published annually since the exam was introduced. Factors influencing college SPLE pass rates were predominantly associated with individual students, including Grade Point Average (GPA) and General Aptitude Test (GAT) scores (6). Other less significant factors include high school results, GPA in Pharmacology courses, GPA in Therapeutics, sex, the year the college was established, and the year the exam was administered (7, 12). Previous studies have not evaluated the effect of accreditation/certification of the program on college pass rates. The purpose of this study was to investigate the association between program accreditation status and college SPLE pass rates.

## Methods

### Study design

A cross-sectional retrospective study design was used to examine the impact of accreditation status on the Saudi Pharmacists Licensure Examination (SPLE) pass rates among pharmacy colleges in Saudi Arabia.

### Data collection

Pharmacy colleges' SPLE pass rates are publicly available and were obtained from the SCFHS report 2024. Pharmacy colleges were categorized into five groups according to the mean of their students' SPLE scores, as shown in Table 1. Programs are categorized into five groups based on their SPLE mean score ranges. Category A includes programs with scores of 590 and above, while Category B encompasses those with scores between 565 and 589. Category C covers a mean score range of 530–564, followed by Category D, which includes scores from 505 to 529. Lastly, Category E comprises programs scoring below 505. Pharmacy colleges' national accreditation status was obtained from the ETEC website, American Council for Pharmacy Education (ACPE) status was obtained from the ACPE website, and the Canadian Council for Accreditation of Pharmacy Programs (CCAPP) status was obtained from the CCAPP website. ACPE International- Accreditation A program is granted international accreditation when, following an on-site initial evaluation, it satisfactorily demonstrates compliance with the Eligibility and Quality Criteria set by the Board. This accreditation indicates reasonable assurance of ongoing compliance with these standards; if the evaluation is conducted online, the program's status will reflect “(online evaluation).” Accredited programs must continuously adhere to the Quality Criteria to maintain their status. Similarly, for programs selected for CCAPP accreditation, a site review may be conducted to assess eligibility for an accreditation award. This award is determined through a comprehensive process that includes an application, a complete self-assessment based on the CCAPP International Accreditation Standards and Guidelines for the First Professional Degree in Pharmacy Programs (2017, revised July 2020), an on-site visit, a written report, and a final decision by the CCAPP Board regarding the accreditation award.

**Table 1:** University SPLE pass score, accreditation status, and institution type.

**University**	*N* ^*^	**Mean**	**Margin of error**	**Confidence interval**		**Ranking category**	**ETEC accreditation**	**International-accreditation**	**Dual accreditation**	**Institution type**
				**Lower limit**	**Upper limit**					
Imam Abdulrahman Bin Faisal University	214	607.83	6.86	600.98	614.69	Category A	Yes	No	No	Government
King Saud Bin Abdulaziz University For Health Sciences	201	602.03	6.89	595.14	608.92	Category A	Yes	No	No	Government
King Saud University	405	600.92	5.60	595.32	606.52	Category A	Yes	Yes/ACPE	Yes	Government
Princess Nourah Bint Abdulrahman University	143	597.68	8.21	589.46	605.89	Category A	Yes	No	No	Government
King Faisal University	188	594.44	7.92	586.51	602.36	Category A	Yes	Yes/ACPE, CAPP	Yes	Government
Alfaisal University	33	586.55	21.35	565.20	607.89	Category B	Yes	No	No	Private
Najran University	22	584.00	18.54	565.46	602.54	Category B	No	No	No	Government
Taibah University	209	582.79	7.83	574.96	590.63	Category B	No	Yes, ACPE	No	Government
King Abdulaziz University	338	581.12	7.08	574.04	588.20	Category B	No	Yes, CCAPP	No	Government
Umm Al-Qura University	368	579.34	5.96	573.38	585.29	Category B	Yes	No	No	Government
Qassim University	391	571.26	5.02	566.25	576.28	Category B	Yes	Yes, ACPE	Yes	Government
University of Tabuk	121	568.93	9.73	559.19	578.66	Category B	Yes	No	No	Government
Taif University	417	560.75	5.28	555.47	566.02	Category C	Yes	Yes, ACPE	Yes	Government
University of Hail	251	550.84	7.68	543.16	558.53	Category C	Yes	No	No	Government
Jazan University	544	547.31	5.44	541.87	552.74	Category C	Yes	No	No	Government
Ibn Sina National College for Medical Studies	278	544.78	7.75	537.03	552.53	Category C	No	Yes, ACPE	No	Government
Northern Borders University	132	537.73	11.44	526.29	549.17	Category C	No	No	No	Government
Al-Baha University	161	534.02	8.50	525.52	542.53	Category C	Yes	No	No	Government
Batterjee Medical College	140	530.04	9.37	520.67	539.41	Category C	No	Yes, ACPE	No	Private
Almaarefa University	185	523.25	7.88	515.38	531.13	Category D	Yes	No	No	Private
King Khalid University	914	521.31	3.90	517.40	525.21	Category D	No	No	No	Government
Shaqra University	186	519.55	11.59	507.96	531.13	Category D	No	No	No	Government
Prince Sattam Bin Abdulaziz University	697	509.31	4.73	504.59	514.04	Category D	No	Yes, ACPE	No	Government
Mohammed Al-Mana College For Medical Sciences	286	508.60	7.16	501.44	515.76	Category D	No	No	No	Private
Buraydah Colleges	214	499.66	7.96	491.70	507.63	Category E	No	No	No	Private
Jouf University	399	494.62	5.71	488.90	500.33	Category E	Yes	No	No	Government
Riyadh Elm University	109	493.28	14.39	478.89	507.67	Category E	No	No	No	Private

N^*^ indicated number of students per college.

#### Data analysis

##### *T*-test analysis

An independent samples *t*-test was conducted to further confirm the findings. The independent samples *t*-test was used to assess the association between colleges' SPLE mean scores and the following dependent variables: accreditation/certification of pharmacy programs (either national or international), national accreditation (ETEC), colleges with international accreditation (ACPE International-Accreditation or CCAPP accreditation), dual accreditation (i.e., national and international), more than one accreditation, and “institution type,” referring to whether a college is private or government-funded (public). The Statistical Package for Social Sciences (SPSS) for Windows version 22 was used.

## Results

**Figure 1:**
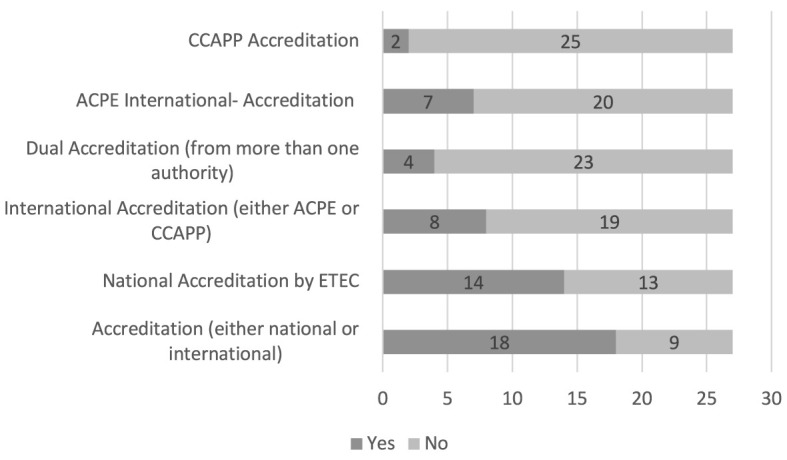
Accreditation status of programs: overview of national and international accreditations.

A total of 27 pharmacy colleges representing 7,546 students were included in the analysis. Twenty-two were government and five were private colleges. Fifteen programs obtained national accreditation by ETEC, nine obtained International-Accreditation from ACPE or CCAPP, and four programs obtained dual accreditation, i.e., both national and international. The categorization of SPLE pass rates was as follows: five were in Category A, seven were in Category B, another seven were placed in Category C, five were in Category D, and three were in Category E (Table 1 and Figure 1).

### The independent samples *t*-test

An independent samples *t*-test was conducted to evaluate colleges' SPLE pass rates with the following variables:

1. **Accreditation (either national or international):** On average, colleges that were accredited by either national (ETEC) or international (ACPE, CCAPP) organizations performed better in SPLE (M = 563, SE = 43.4) than those that were not accredited (M = 533, SE = 33.6). This difference was significant [*t*_(22)_ = −2.149, *p* = 0.042].
2. **National accreditation by ETEC:** On average, colleges that obtained ETEC accreditation achieved better scores in SPLE (M = 569.2, SE = 44.8) than those that were not accredited (M = 535.6, SE = 31.6). This difference was significant [*t*_(25)_ = −2.7, *p* = 0.013].
3. **International accreditation (either ACPE or CCAPP):** On average, colleges with international accreditation (ACPE and/or CCAPP) scored higher in SPLE (M = 561.5, SE = 31.9) than those that were not accredited (M = 561.02, SE = 31.9). This difference was not significant [*t*_(25)_ = −0.7, *p* = 0.4].
4. **Dual accreditation (from more than one authority):** On average, colleges that were awarded accreditation from more than one authority performed better in SPLE (M = 581.8, SE = 18.9) than those that were not accredited by two or more authorities (M = 548.02, SE = 18.9). This difference was not significant [*t*_(25)_ = −1.8, *p* = 0.086].
5. **ACPE International-Accreditation:** On average, colleges that obtained ACPE International- Accreditation performed better in SPLE (M = 558.8, SE = 33.4) than those that were not accredited (M = 551, SE = 38). This difference was not significant [*t*_(25)_ = −0.48, *p* = 0.63].
6. **CCAPP accreditation:** On average, colleges that obtained CCAPP accreditation performed better in SPLE (M = 587.8, SE = 9.5) than those that were not accredited (M = 550.3, SE = 36.4). This difference was not significant [*t*_(25)_ = −1.4, *p* = 0.17].
7. **Institution type (government vs. private):** On average, government-funded colleges performed better in SPLE (M = 562.3, SE = 33.8) than private colleges (M = 526.6, SE = 31.9). This difference was significant [*t*_(25)_ = −2.1, *p* = 0.022].

## Discussion

The pharmacy profession in Saudi Arabia has gone through tremendous changes in the last decade. There has been a growing number of entry-level pharmacy education programs, from 1 in 2001 to 30 in 2014 (8), an increasing number of private pharmacy institutions, and a rising number of pharmacy graduates who have started replacing the international pharmacy workforce (9). All these transformations have positively impacted the workforce capacity (3, 10). However, these changes have raised concerns about the quality of pharmacist students' education.

Therefore, recent initiatives have shifted focus from the availability of pharmacy professionals toward enhancing the quality of education, training, and readiness to practice. Pharmacy colleges within well-established academic institutions such as King Saud University and King Abdulaziz University pursued international accreditation for their entry-level professional degrees. Other colleges adopted the trend before ETEC required local accreditation. Since accreditation became mandatory, other colleges have pursued national accreditation (5). Another quality assurance tool or readiness to practice assessment method was the introduction of the SPLE (SCFHCS, 2024).

Considering that the accreditation of pharmacy programs and licensing examinations are the two major quality standards for pharmacists' readiness to practice in Saudi Arabia, this study was conducted to evaluate the impact of accreditation on examination pass rates.

It was evident that accreditation, whether national or international, significantly enhanced SPLE scores, with pharmacists who graduated from accredited institutions performing better than those from institutions that did not obtain accreditation.

Another finding is that national accreditation by ETEC resulted in significantly higher SPLE exam grades, whereas international accreditation also led to higher scores, but the impact was not significant. The same was observed with dual accreditation (national and international), ACPE International-Accreditation, and CCAPP accreditation. Although these led to higher SPLE scores, the effect was not significant.

The findings raise the question of whether publicly funded pharmacy institutions need to obtain international accreditation, given that most of them, i.e., King Saud University, King Faisal University, Taif University, and Qassim University, achieved national accreditation, except for King Abdulaziz University, which has only received accreditation from CCAPP, and Taibah University, which has only received ACPE International-Accreditation.

Additionally, the top two colleges, Imam Abdulrahman Bin Faisal University and King Saud Bin Abdulaziz University for Health Sciences, in terms of SPLE pass rates, only have national accreditation, further supporting the evidence that international accreditation does not necessarily enhance pharmacists' readiness to practice within a local context.

Another point to highlight is that government-funded universities, particularly the reputable ones, do not necessarily need dual or international accreditation. Even though such accreditation enhances their reputation, these institutions attract high-quality students regardless. This may not be the case for private institutions, which are competing to attract students to their programs.

Comparing the SPLE results of government and private institutions confirmed that government colleges performed better. Only one private college, Faisal University, was placed in Category B, while the other private colleges were in Categories C, D, and E. On the other hand, although Najran University, a government institution, does not have accreditation, it was still placed in a higher category. This finding highlights the confounding variables related to individual students, which were previously addressed in other studies (6, 12) and are beyond the scope of this research. Generally speaking, private pharmacy education in Saudi Arabia tends to attract less competent students, which may affect their SPLE pass rates regardless of their accreditation status.

Regulation of pharmacy education quality through accreditation and licensing examinations is relatively new (8, 11). The involvement of two organizations, ETEC and SCFHS, with overlapping responsibilities and approaches, may impact the efficiency of achieving desired quality outcomes.

For example, SCFHS requires applicants for the SPLE to have graduated from an accredited institution. However, students from non-accredited institutions are still allowed to take the exam. It might be considered unreasonable to disqualify students from taking the licensing examination solely because their college had not yet been accredited.

This transitional period is likely to resolve as more programs achieve accreditation each year. There are now 18 accredited programs compared to 9 in 2021 (17).

This investigation has some limitations. The dependent variable was colleges' average SPLE scores. Using data from a single year may limit the interpretation of trends or findings from previous years. However, this dataset was chosen for the following reasons: it directly reflects the impact of accreditation status on SPLE scores, which is the primary focus of this research; many colleges have only recently obtained accreditation; and finally, the data is produced by the SCFHS and is publicly available.

There are two implications from this investigation. One, further research is needed to examine individual student factors that contribute to the exam results of the top-rated colleges. Stakeholders involved in regulating the quality of pharmacy education, such as ETEC, can play a critical role in supporting programs that are not currently accredited. Support and guidance could be provided through advisory services, workshops and training sessions, resource provision, continuous monitoring and feedback, and collaboration with accredited institutions.

## Conclusion

Accredited pharmacy programs demonstrated higher SPLE pass rates. National accreditation by ETEC appeared to be sufficient to meet national regulations and improve SPLE pass rates. Dual accreditation or international accreditation did not seem necessary for enhancing SPLE exam results and pharmacists' readiness to practice.

## Data Availability

The original contributions presented in the study are included in the article/supplementary material, further inquiries can be directed to the corresponding author.
